# Formation of an unexpected 3,3-diphenyl-3*H*-indazole through a facile intramolecular [2 + 3] cycloaddition of the diazo intermediate

**DOI:** 10.3762/bjoc.15.134

**Published:** 2019-06-19

**Authors:** Andrew T King, Hugh G Hiscocks, Lidia Matesic, Mohan Bhadbhade, Roger Bishop, Alison Thavary Ung

**Affiliations:** 1 School of Mathematical and Physical Sciences, University of Technology Sydney, PO Box 123, Broadway, NSW, 2007, Australia; 2 Australian Nuclear Science and Technology Organisation, Locked Bag 2001, Kirrawee DC, NSW, 2232, Australia; 3 Mark Wainwright Analytical Centre, University of New South Wales, Sydney, NSW, 2052, Australia,; 4 School of Chemistry, University of New South Wales, Sydney, NSW, 2052, Australia

**Keywords:** Ar-H^...^H-Ar contact, [2 + 3] cycloaddition, diazo, 3*H*-indazole, X-ray structure

## Abstract

The one-pot reaction of 2,6-bis(diphenylmethyl)-4-methoxyaniline with *tert*-butylnitrite, BTEAC and DABSO in the presence of CuCl_2_ provided an unexpected 3*H*-indazole product **8**. The structure of the compound was determined by HRMS, IR, NMR and further confirmed by single crystal X-ray crystallography. The compound crystallises in the triclinic *P*-1 space group, with unit cell parameters *a* = 9.2107 (4), *b* = 10.0413 (5), *c* = 14.4363 (6) Å, α = 78.183 (2), β = 87.625 (2), γ = 71.975 (2)°. The formation of **8** proceeded through a facile intramolecular [2 + 3] cycloaddition of the diazo intermediate **9**. The molecules of **8** are organised by edge–face Ar–H···π, face–face π···π, and bifurcated OCH_2_–H···N interactions. In addition to these, there are Ar–H···H–Ar close contacts, (edge–edge and surrounding inversion centres) arranged as infinite tapes along the *a* direction.

## Introduction

The use of sulfonyl fluorides in biological applications has been on the increase as evidenced by the number of recent publications [1–3]. One potential application of sulfonyl fluorides is as an ^18^F-radiolabelled synthon that can be conjugated to macromolecules and used to image cancer and other diseases in the body. Synthons are becoming more prevalent in imaging modalities like positron emission tomography (PET) as there is a greater need to further investigate lesions, and disease tissues on the molecular level. However, issues with the stability of sulfonyl fluorides have been reported in the literature [4–5].

**Figure 1:**
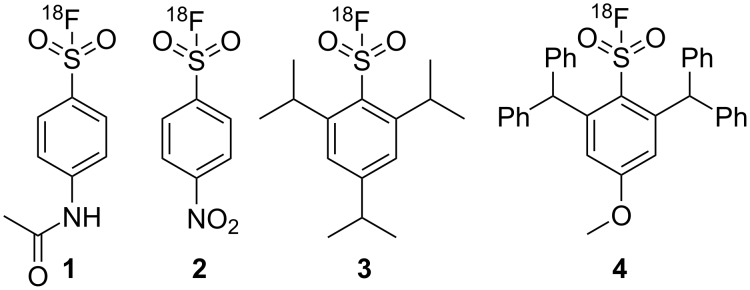
Examples of ^18^F-radiolabelled arylsulfonyl fluorides containing electron-donating **1**, electron-withdrawing **2**, and sterically bulky groups **3** [5] and **4**.

Stability and radiochemical improvements were investigated by Matesic et al. [5]. For instance, electron-donating ^18^F-arylsulfonyl fluoride **1** was >98% stable in the buffer at 3 h, while the electron-withdrawing compound **2** was only 8% stable [5] (Figure 1). The more sterically hindered ^18^F-arylsulfonyl fluoride **3** was 95% intact in rat plasma after 120 min at 37 °C [5]. This indicated that the combination of electron-donating effects and steric hindrance of isopropyl groups provide greater stability to a sulfur–fluorine bond than groups that can only provide the steric hindrance in the molecule [6].

**Scheme 1:**
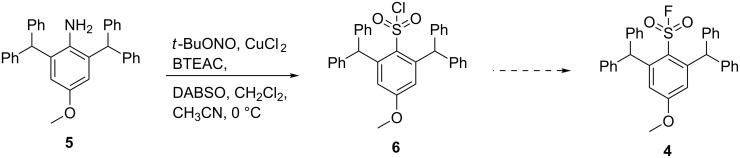
Reaction for the formation of sulfonyl chloride **6** using DABSO.

To further investigate the stability of ^18^F-sulfonyl fluorides, the sterically hindered compound **4** was selected with enough steric bulk in the 2,6-position. The initial chlorosulfonylation reaction was performed using 1,4-diazabicyclo[2.2.2]octane bis(sulfur dioxide) adduct (DABSO) as a source of sulfur dioxide. DABSO was selected as it would provide a facile method and is a safer alternative to sulfur dioxide gas [7–10]. The target molecule **4** was envisaged via the diazonium salt of **5** [11] and **6** and was a modification of the microfluidic flow reaction reported by Malet-Sanz et al. [12] (Scheme 1).

Herein, we report the unexpected product derived from **5** under the reaction conditions described in Scheme 1. The unusual crystal packing present in this 3*H*-indazole product was also analysed.

## Results and Discussion

**Figure 2:**
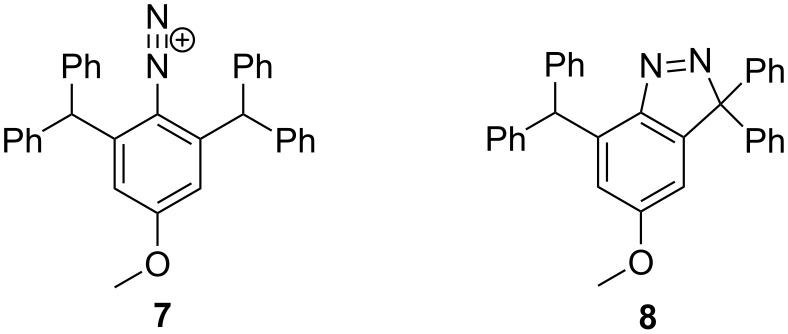
Possible compounds with the molecular formula C_33_H_26_N_2_O (structure **7** contains 27 hydrogen atoms).

The one-pot reaction of compound **5** with *tert*-BuONO and DABSO in the presence of benzyltriethylammonium chloride (BTEAC) and CuCl_2_ gave a crude product which was purified by crystallisation from dichloromethane/hexane to give a pale yellow crystalline material in 27% yield. HRMS–ESI analysis produced [M + H]^+^ *m/z* 467.2126 corresponding to the formula of C_33_H_27_N_2_O. Considering that the expected mass of the sulfonyl chloride **6** was *m/z* 539.1403 with the corresponding formula of C_33_H_28_ClO_3_S, it was concluded that **6** did not form. It was initially thought that the product was the diazonium intermediate **7** (Figure 2), as it was expected from the reaction conditions and the HRMS analysis of diazonium ion **7** in the form of [M]^+^ was C_33_H_27_N_2_O^+^.

### Spectroscopy analysis

A close analysis of the ^1^H NMR spectrum revealed the non-symmetrical substitution of the aromatic core. Two aromatic protons appeared as two doublets (*J* = 2 Hz) at 6.92 and 6.67 ppm, respectively. Furthermore, the spectrum showed only one benzylic proton, as a singlet at 6.82 ppm. ^13^C NMR and HSQC showed tertiary and quaternary resonances at 51.3 and 101.5 ppm, respectively. The first resonance is indicative of HC(Ph)_2_, while the one at 101.5 ppm is more likely to be the 3,3-diphenyl-substituted carbon of the indazole ring. The ambiguity was overcome through the X-ray structural analysis of the compound. The single crystals were easily obtained by recrystallisation from CH_2_Cl_2_.

### Crystal structure analysis

**Figure 3:**
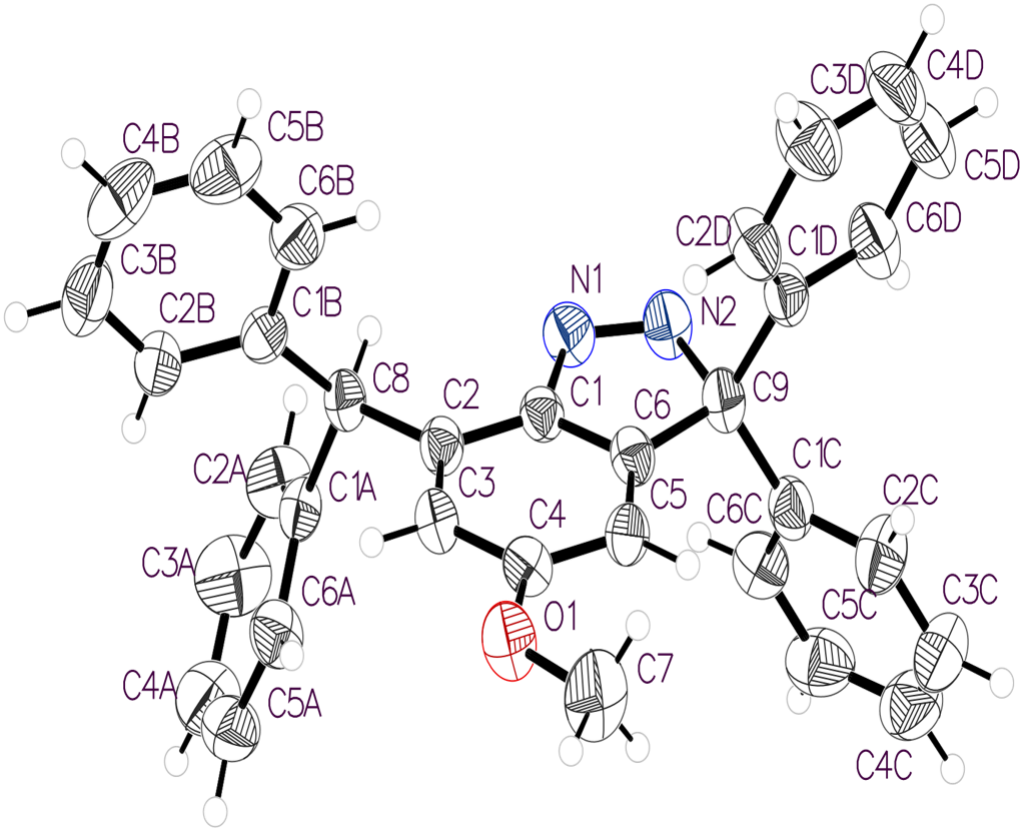
ORTEP view of the molecule **8** showing the atom labelling (ellipsoids are drawn at 50% probability level).

The X-ray crystal analysis revealed that the product was not the diazonium intermediate **7**, but rather the compound **8** containing the 3,3-diphenyl-3*H*-indazole core structure. The crystals were found to be triclinic, *P*-1 space group with cell constants *a* = 9.2107(4), *b* = 10.0413(5), *c* = 14.4363(6) Å, α = 78.183(2), β = 87.625(2), γ = 71.975(2)°. The structure of **8** and the atom-labelling scheme are presented in Figure 3. The crystal and refinement data are given in Table 1. The 3*H*-indazole **8** (–N=N–CRR–) has a break in its conjugation at the sp^3^ carbon atom when it is compared to the 1*H*-indazole isomer –NH–N=CH– that is fully conjugated with the benzo group. Therefore, the 3*H*-indazole isomer is higher in energy and thus much less common. The bond lengths within this 3*H*-indazole ring structure confirm the evidence for sp^2^ N=N. The N1–C1 and N2–C9 distances are 1.426(2) Å and 1.530(2) Å. The N1=N2 length was found to be 1.266(2) Å, a value that agrees with literature reports [13].

**Table 1:** Crystal data and structure refinement for compound **8**.

**crystal data**	

chemical formula	C_33_H_26_N_2_O
*M*_r_	466.56
crystal system, space group	triclinic, *P*-1
temperature (K)	150
*a*, *b*, *c* (Å)	9.2107 (4), 10.0413 (5), 14.4363 (6)
α, β, γ (°)	78.183 (2), 87.625 (2), 71.975 (2)
*V* (Å^3^)	1242.40 (10)
*Z*	2
radiation type	Mo Kα
µ (mm^−1^)	0.08
crystal size (mm)	0.22 × 0.19 × 0.11

**data collection**	

diffractometer	Bruker *APEX*-II CCD
absorption correction	–
no. of measured, independent and observed [*I* > 2σ(*I*)] reflections	40887, 5423, 4236
*R*_int_	0.039
(sin θ/λ)_max_ (Å^−1^)	0.640

**refinement**	

*R*[*F*^2^ > 2σ(*F*^2^)], *wR*(*F^2^*), *S*	0.054, 0.157, 1.04
no. of reflections	5423
no. of parameters	326
H-atom treatment	H-atom parameters constrained
Δρ_max_, Δρ_min_ (e Å^−3^)	0.51, −0.20
CCDC deposition number	1902859

**Figure 4:**
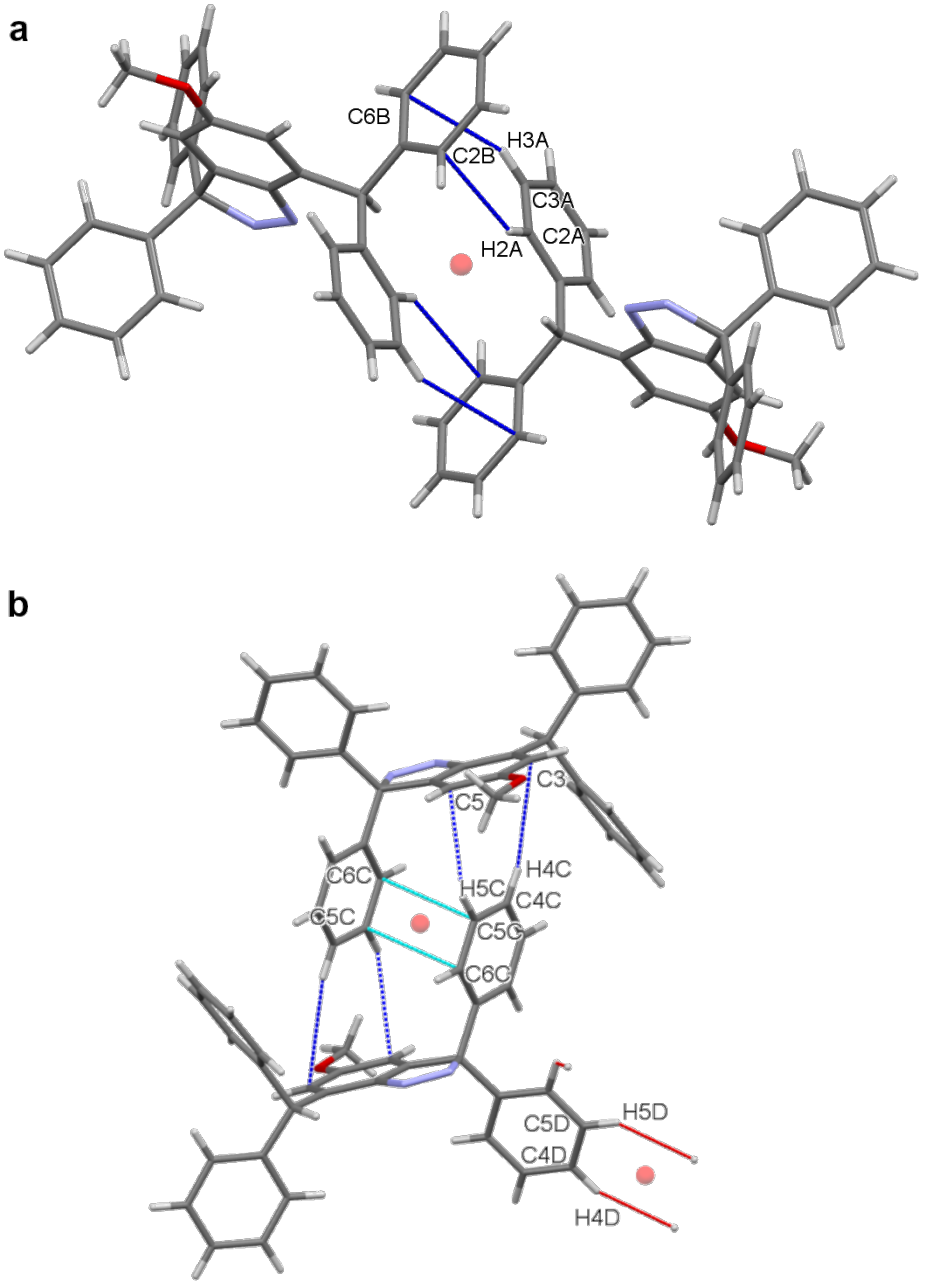
Significant intermolecular interactions made by the benzhydryl group (a, upper) and the *gem*-diphenyl group (b, lower). Crystallographic colour code: C–H···π (dark blue), π···π (light blue), inversion centre (orange sphere), and incomplete contacts (red).

The 3,3-diphenyl-3*H*-indazole core of **8** carries both benzhydryl and germinal diphenyl groups. These aromatic substituents make significantly different intermolecular contacts in the crystal. The phenyl rings of the former (C1A to C6A, and C1B to C6B) make complementary C–H···π interactions with the related molecule across a centre of inversion. Hydrogen atoms C2A–H2A and C3A–H3A make an almost perpendicular approach to ring B at C2B and C6B, respectively: H2A···C2B = 3.04 Å and H3A···C6B = 2.96 Å (Figure 4a). One of the *gem*-diphenyl rings (C1C to C6C) also employs C-H···π interactions across a centre of inversion. Its partner is, however, the aromatic ring of the core (C1 to C6). Thus, the hydrogens C5C–H5C and C4C–H4C make nearly perpendicular approaches to atoms C5 and C3, respectively: H5C···C5 = 2.95 Å and H4C···C3 = 3.08 Å. This phenyl ring also participates in a slipped π···π contact with its inversion-related counterpart: C6C···C5C = 3.50 Å (Figure 4b).

**Figure 5:**
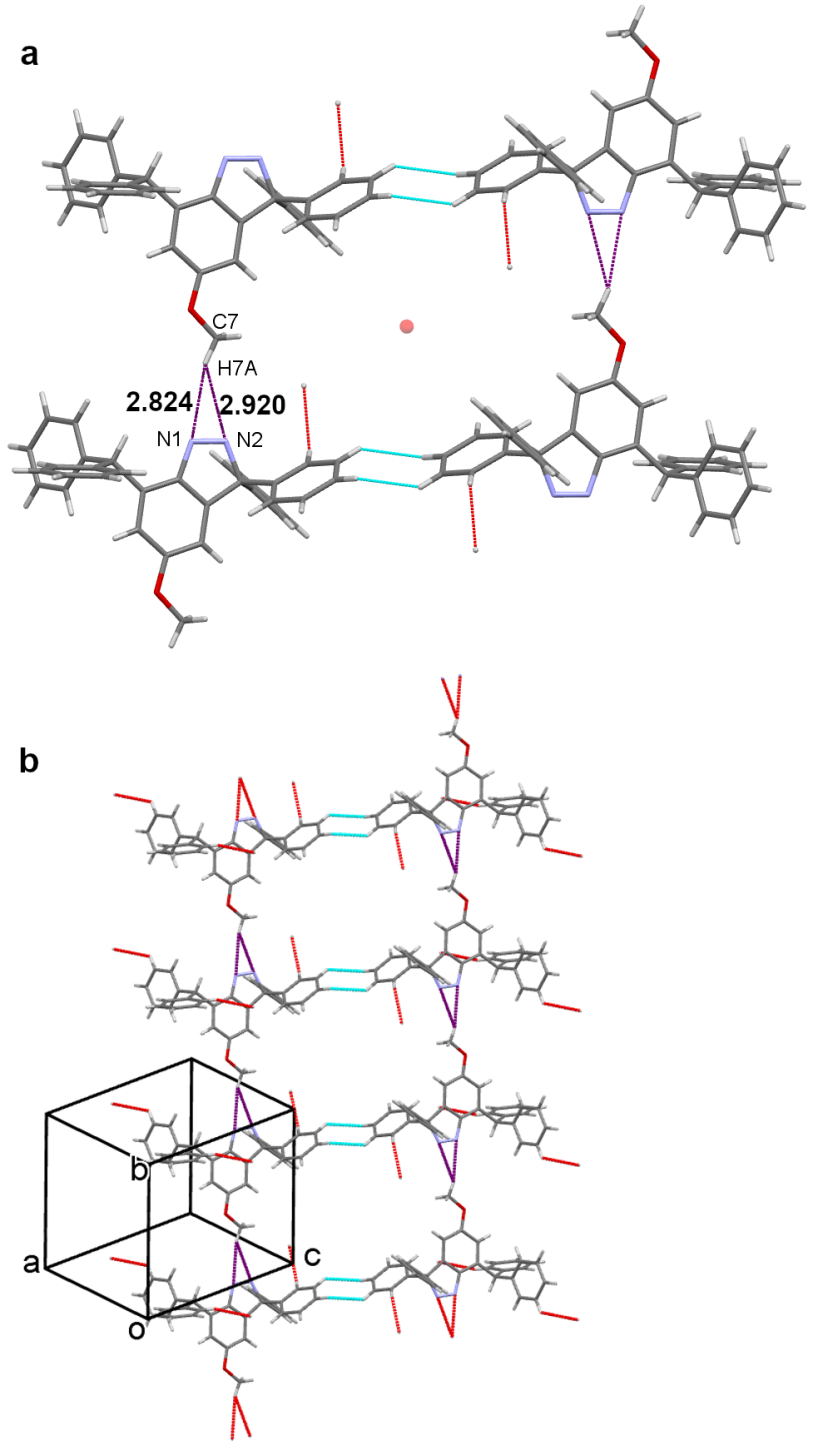
Relationship of the C–H···N and cyclic C–H···H-C contacts in the crystal structure of **8**. The centrosymmetric array of four molecules (**5a**, upper) and a stepladder association of eight molecules (**5b**, lower). Crystallographic colour code: C–H···N purple), C–H···H–C (light blue), inversion centre (orange sphere), and incomplete contacts (red).

The C4 methoxy and the heterocyclic diazo functionality of **8** are linked through an interesting arrangement along the *b* axis that can be described either as a OCH_2_–H···π (N=N) or as a bifurcated N···H(CH_2_O)···N interaction. This motif is close to symmetrical with H···N distances of C7–H7A···N1 = 2.82 Å and C7–H7A···N2 = 2.92 Å. The second of the *gem*-diphenyl rings (C1D to C6D) surrounds an inversion centre along with its counterpart through a cycle of two C4D–H4D···H5D–C5D contacts. Four molecules of **8**, involving two C–H···N and two cyclic H···H motifs, surround another inversion centre (Figure 5a). Consideration of additional molecules reveals a stepladder assembly along *b*, in which the C–H···N interactions form the side rails and the cyclic H···H motifs the rungs (Figure 5b).

**Figure 6:**
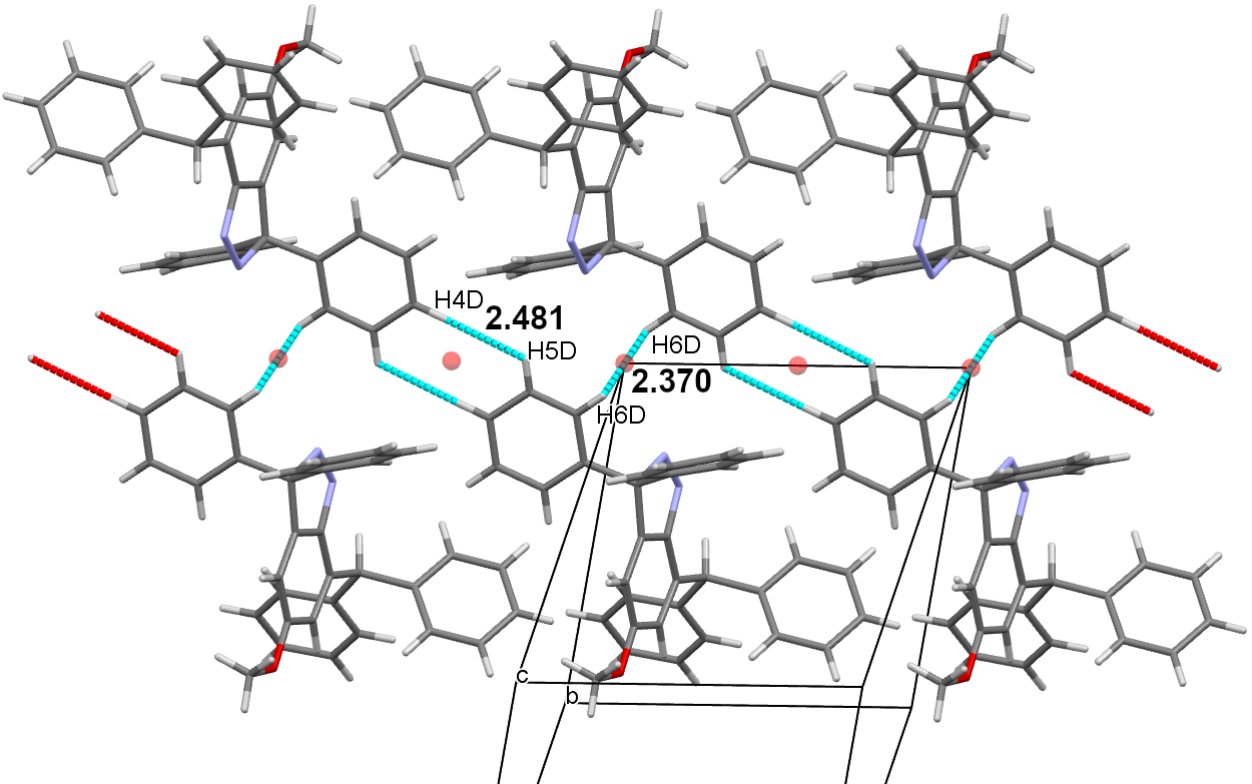
Part of a hydrocarbon tape along *a* formed by a combination of alternating linear and cyclic C–H···H–C close contacts. Crystallographic colour code: C–H···H–C (light blue), inversion centre (orange sphere), and incomplete contacts (red).

As well as the centrosymmetric cycle of C4D–H4D···H5D–C5D contacts (H···H = 2.48 Å) there is also a linear C6D–H6D···H6D–C6D close contact (H···H = 2.37 Å) around a further inversion centre. These two motifs alternate along the *a* axis to produce infinite hydrocarbon tapes with inversion centres separated by *a*/2 (Figure 6).

The molecular structure of **8** precludes Pauling-type hydrogen bonding, and therefore weaker attractions must be used in the crystal packing. Its interaction between the methoxy and diazo functionalities is uncommon, though entirely reasonable in the context of weak hydrogen bonding [14]. The presence of close C–H···H–C contacts in this structure was, however, unexpected and required careful consideration. First, one of us has reported previously a short C–H···H–C contact in another structure [15]. This was a linear centrosymmetric motif formed by Ar–H (H···H = 2.35 Å) and is directly analogous in structure to the linear example (2.37 Å) found here. Both distances are less than the van der Waals (VDW) separation of 2.40 Å [16]. The new cyclic example is slightly longer (2.48 Å), but well under the value of 2.80 Å (= VDW + 0.40) discussed by Dance [17]. All three distance values must be treated with caution since their H atoms are in calculated positions. However, since these are well-defined Ar–H groups, their positional errors will be relatively small. Over recent years much understanding has been gained of dihydrogen bonding X–H···H–Y [18–19]. Close examination has been made of the alkane C–H···H–C contact, particularly for reactive structures stabilised by multiple *tert-*butyl groups [20–21], multi-ring cage hydrocarbons [22–23], and linear alkanes [22–23]. The interaction was found to be attractive in all these cases, and computational justifications have been published [21,23]. It, therefore, appears probable that, despite their unfamiliarity, the C–H···H–C contacts illustrated in Figure 6 are similarly attractive in nature.

### Proposed mechanism for the formation of **8**

Indazoles are well-recognised for their important biological activities. They are known to be used as building blocks in drug development. A review by Gaikwad et al. describes reliable routes to particularly fused aromatic 1*H* and 3*H*-indazoles [24]. The common synthetic routes for the formation of cyclic 1*H*-indazoles are diazotisation of corresponding *o*-alkylanilines [25] and nitrosation of the *N*-acetyl derivatives of 2-alkylanilines (Jacobson modification) [26–28]. More recently, the formation of cyclic 3,3-disubstituted 3*H*-indazoles was reported to form mainly through the [2 + 3] cycloaddition of diazo compounds with arynes under mild reaction conditions [29–31]. However, none of these contained a 2-diphenylmethyl (benzhydryl) aniline system, as found in our compound **5**.

**Scheme 2:**
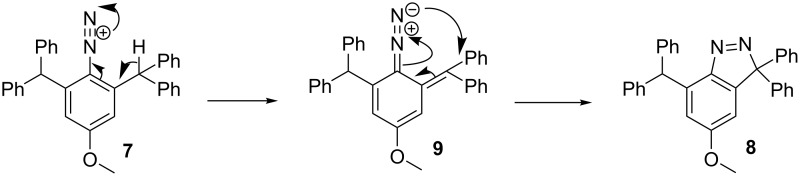
Proposed mechanism for the formation of **8**.

The formation of compound **8** can be explained by the rearrangement of **7** through a hydrogen-shift to form the diazo intermediate **9** which underwent a concerted [2 + 3] intramolecular cyclisation to 3,3-diphenyl-3*H*-indazole **8** (Scheme 2). The process was facilitated by the presence of a highly activated benzylic carbon and further expedited by three phenyl groups, to form the resonance stabilisation of **9** [32]. Furthermore, **8** cannot attain planarity of the methylene–benzhydryl fragment due to steric hindrance. These reactions appear to be also encouraged by the reduction of the crowding originally present in the diazonium salt.

**Scheme 3:**
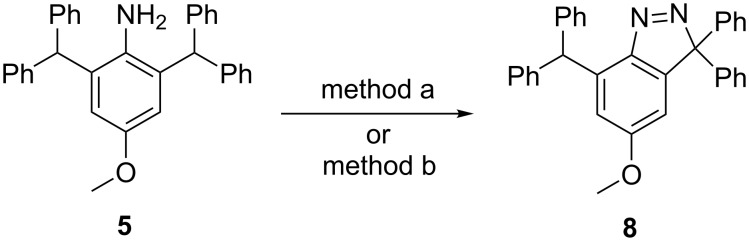
Direct preparation of compound **8**. method a: *t*-BuONO, CuCl_2_, dry CH_3_CN, −10 °C, 89%; method b: NaNO_2_, HCl/acetic acid, −10 °C, 96%.

The direct diazotisation of **5** was performed using anhydrous organic solvents and *t*-BuONO at −10 °C in the presence of CuCl_2_ (method a) and the classical NaNO_2_/HCl method b in the absence of CuCl_2_, as shown in Scheme 3. Compound **8** was obtained in 89% and 96% from method a or b, respectively. The results confirm our initial hypothesis that the cyclisation occurred through the diazo species **9** (Scheme 2) and the Cu^2+^ plays no part in the reaction process.

## Conclusion

The formation of the unexpected product **8** from **5** under anhydrous diazotisation conditions has led to the discovery of a facile synthetic pathway to 3,3-diphenyl-*3H*-indazoles. Formation of **8** has been proposed to proceed through the diazonium intermediate. This was further confirmed by reactions of **5** using *t*-BuONO, and the conventional NaNO_2_/HCl method to give **8** in almost quantitative yields. The crystal structure of **8** involves edge–face Ar–H···π, face–face π···π, bifurcated OCH_2_–H···N, plus edge–edge centrosymmetric Ar–H···H–Ar interactions. These rather uncommon examples of dihydrogen contact assemble as infinite tapes along the *a* direction.

## Experimental

### Materials and physical measurements

All chemical reagents and analytical grade solvents were obtained from commercial sources such as Sigma-Aldrich, Cambridge Isotope Laboratories Inc. and Merck Millipore. All reactions were monitored using either TLC aluminium oxide 60 F254 neutral or TLC Silica gel 60 F254 with UV detection at 254 nm. ^1^H NMR and ^13^C NMR spectra were recorded on an Agilent 500 MHz spectrometer (500 MHz ^1^H, 125 MHz ^13^C) in deuterated chloroform (CDCl_3_). The mass spectral data were obtained using the attached Agilent 5973n MS (EI) spectrometer. High-resolution mass spectra were obtained using an Agilent 6510 Q-TOF Mass Spectrometer (ESI). The infrared spectra were recorded on an Agilent Cary 630 FTIR with a diamond window using 16 background and sample scans. Melting points were measured on a Gallenkamp Melting Point Apparatus equipment and were uncorrected.

### Synthesis of 7-benzhydryl-5-methoxy-3,3-diphenyl-3*H*-indazole (**8**)

2,6-Bis(diphenylmethyl)-4-methoxyaniline (**5**, 0.23 g, 0.505 mmol, 1 equiv) DABSO (0.57 g, 2.37 mmol, 4.75 equiv) and BTEAC (0.12 g, 1 mmol, 1 equiv) were dissolved in DCM (5 mL, solution A) that had been cooled to 0 °C. *tert*-Butyl nitrite (0.15 g, 170 μL) was dissolved in DCM (6 mL) and cooled to 0 °C (solution B). Copper(II) chloride (0.02 g, 0.10 mmol, 0.1 equiv) was dissolved in acetonitrile (6 mL) with sonication and cooled to 0 °C (solution C). Once all solutions had been cooled the following order of addition was used: solution C was added to solution A dropwise. Solution B was added dropwise to solutions A and C. The reaction was allowed to react at room temperature for 24 h before the organic product was extracted using DCM (3 × 10 mL) and brine (10 mL). The organic layers were combined, dried over anhydrous sodium sulphate and the solvent was removed under reduced pressure. The solid crude product was crystallised from dichloromethane to give a brown crystalline solid (0.064 g, 0.137 mmol, 27%); mp 183–184 °C; IR (neat) *v*_max_/cm^−1^: 3057, 3025, 2959, 2926, 2650, 2322, 2112, 1943, 1591, 1490, 1470, 1445, 1349, 1263, 1205, 1127, 1023, 843, 748, 607; ^1^H NMR (500 MHz, CDCl_3_) δ 7.28–7.31 (m, H-Ar, 10H), 7.26–7.21 (m, H-Ar, 10H), 6.92 (d, *J* = 2.0 Hz, H-Ar, 1H), 6.82 (s, *H*C(Ph)_2_, 1H), 6.67 (d, *J* = 2.0 Hz, H-Ar, 1H), 3.75 (s, O-C*H*_3_, 3H); ^13^C NMR (125 MHz, CDCl_3_) δ 161.6 (C), 149.9 (C), 146.6 (C), 142.9 (2C), 139.9 (C), 138.3 (2C) 129.7 (4CH), 128.6 (4CH), 128.4 (4CH), 128.0 (2CH), 127.8 (4CH), 126.6 (2CH), 115.9 (CH), 107.7 (CH), 101.5 (*C*(Ph)_2_), 55.8 (CH_3_), 51.3 (H*C*(Ph)_2_); HRMS–ESI (*m*/*z*): [M + H]^+^ calcd for C_33_H_27_N_2_O, 467.2118; found, 467.2126.

#### Direct preparation of compound **8** - method a

Under a nitrogen atmosphere, compound **5** (0.23 g, 0.505 mmol, 1 equiv) was dissolved in acetonitrile (20 mL) then cooled to 0 °C (solution A). *tert*-Butyl nitrite (0.15 g, 170 μL) was dissolved in DCM (6 mL) and cooled to 0 °C (solution B). Copper(II) chloride (0.02 g, 0.10 mmol, 0.2 equiv), was dissolved in acetonitrile (6 mL) with sonication and cooled to 0 °C (solution C). Once all solutions had been cooled, the following order of addition was used: solution C was added to solution A dropwise. Solution B was added dropwise to solutions A and C. The reaction was allowed to react at room temperature for 24 h before the organic product was extracted using DCM (3 × 10 mL) and brine (10 mL). The organic layers were combined, dried over anhydrous sodium sulphate and the solvent was removed under reduced pressure to give the crude product which was purified by filtering through a short column flash silica gel using DCM as the mobile phase to give **8** (0.210 g, 0.449 mmol, 89%).

#### Direct preparation of compound **8** - method b

Compound **5** (0.23 g, 0.505 mmol, 1 equiv) was dissolved in acetonitrile (10 mL) at room temperature before acetic acid (0.96 mL, 14.76 mmol, 29 equiv) and concentrated hydrochloric acid (0.98 mL, 27 mmol, 54 equiv) was added dropwise to the solution over 2 min at −10 °C. Sodium nitrite (0.123 g, 1.72 mmol, 3.4 equiv) in water (5 mL) was added to the stirring solution over 1 min at −10 °C. The resulting solution was stirred for 1 h at −10 °C followed by 24 h at room temperature. The organic product was extracted using DCM (3 × 20 mL) and brine (30 mL). The organic layers were combined, dried over anhydrous sodium sulphate and the solvent was removed under reduced pressure to give the pure product **8** (0.226 g, 0.485 mmol, 96%). The ^1^H NMR spectrum of the crude product indicates that it is pure and required no further purification.

### X-ray crystallography

A colourless plate-like crystal of **8** with dimensions of 0.11 × 0.19 × 0.22 mm, selected under the polarising microscope (Leica M165Z), was mounted on a MicroMount (MiTeGen, USA) consisting of a thin polymer tip with a wicking aperture. The X-ray diffraction measurements were carried out on a Bruker kappa-II CCD diffractometer at 150 K using IµS Incoatec Microfocus Source with Mo Kα radiation (λ = 0.710723 Å). The single crystal, mounted on the goniometer using a cryo-loop for intensity measurements, was coated with immersion oil type NVH and then quickly transferred to the cold nitrogen stream generated by an Oxford Cryostream 700 series. Symmetry-related absorption corrections using the program SADABS [33] were applied, and the data were corrected for Lorentz and polarisation effects using Bruker APEX3 software [33]. The structure was solved by program SHELXT [34] (with intrinsic phasing), and the full-matrix least-square refinements were carried out using SHELXL-2014 [35] through Olex2 [36] suite of software. The non-hydrogen atoms were refined anisotropically. Molecular graphics were generated using Mercury [37]. Key crystallographic data and refinement details are presented in Table 1.

### Crystal structure data

Crystallographic data (excluding structure factors) for the structures in this paper have been deposited with the Cambridge Crystallographic Data Centre as supplementary publication number 1902859. The data can be obtained free of charge via http://www.ccdc.cam.ac.uk or by e-mailing data_request@ccdc.cam.ac.uk, or by contacting The Cambridge Crystallographic Data Centre, 12, Union Road, Cambridge CB2 1EZ, UK; fax: (+44) 1223/336-033, Tel.: (+44) 1223/336-408.

## Supporting Information

File 1 Copies of ^1^H NMR, ^13^C NMR and IR of compound **8**.
