# Supplementary material for: Formation of an unexpected 3,3-diphenyl-3H-indazole through a facile intramolecular [2 + 3] cycloaddition of the diazo intermediate
Source: Beilstein J Org Chem. 2019 Jun 19;15:1347–54. doi: 10.3762/bjoc.15.134 (PMC6604747; doi:10.3762/bjoc.15.134)
Supplement: File 1 — Copies of 1H NMR, 13C NMR and IR of compound 8. [file Beilstein_J_Org_Chem-15-1347-s001.pdf]

BEILSTEIN JOURNAL OF ORGANIC CHEMISTRY

Supporting Information

for

Formation of an unexpected 3,3-diphenyl-3*H*-indazole through a facile intramolecular [2 + 3] cycloaddition of the diazo intermediate

Andrew T. King, Hugh G. Hiscocks, Lidia Matesic, Mohan Bhadbhade, Roger Bishop and Alison T. Ung

Beilstein J. Org. Chem. **2019,** *15,* 1347–1354. doi:10.3762/bjoc.15.134

Copies of ^{1}H NMR, ^{13}C NMR and IR of compound 8

License and Terms: This is a supporting information file under the terms of the Creative Commons Attribution License (http://creativecommons.org/licenses/by/4.0). Please note that the reuse, redistribution and reproduction in particular requires that the authors and source are credited.
The license is subject to the *Beilstein Journal of Organic Chemistry* terms and conditions: (https://www.beilstein-journals.org/bjoc)

NMR spectra of compound 8

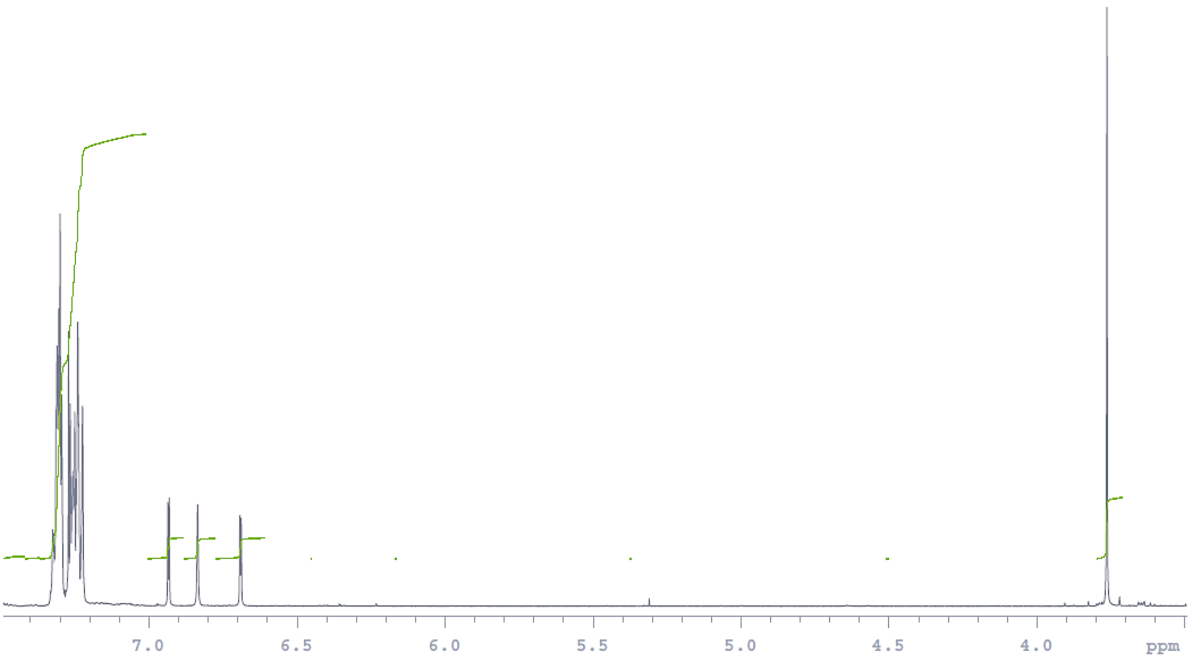

Figure S1: ^{1}H NMR ($CDCl_3$, 500 MHz) of compound **8**.

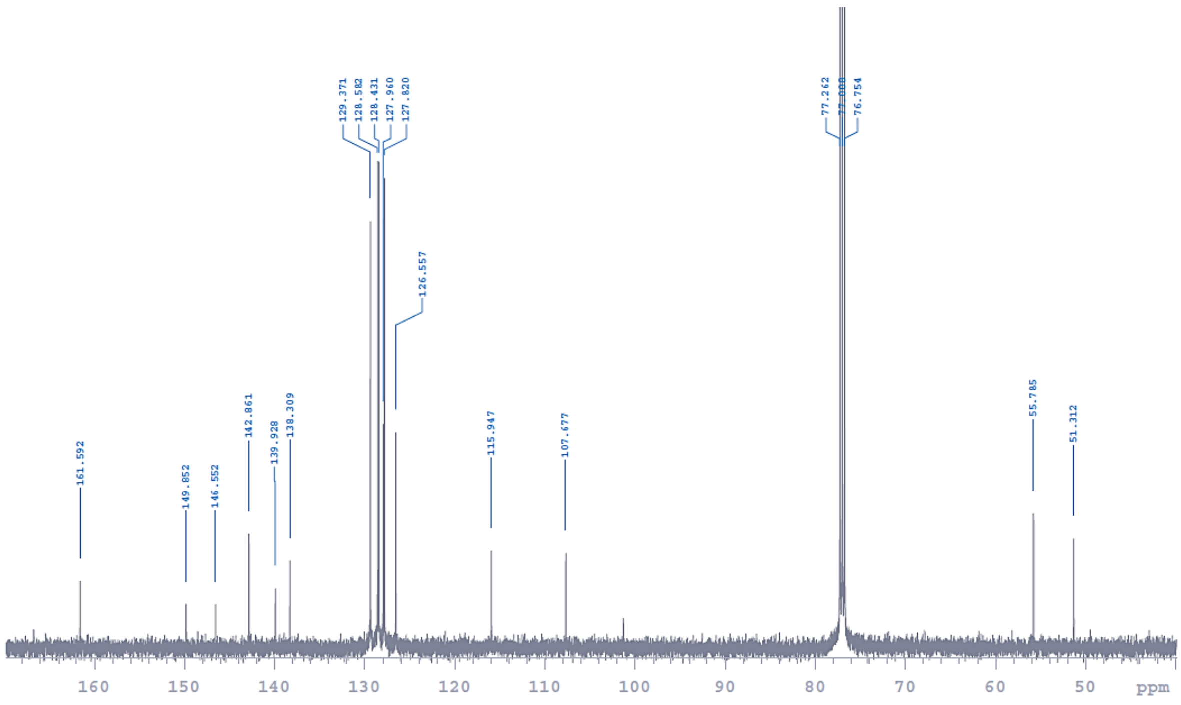

Figure S2: ^{13}C NMR ($CDCl_3$, 125 MHz) of compound **8**.

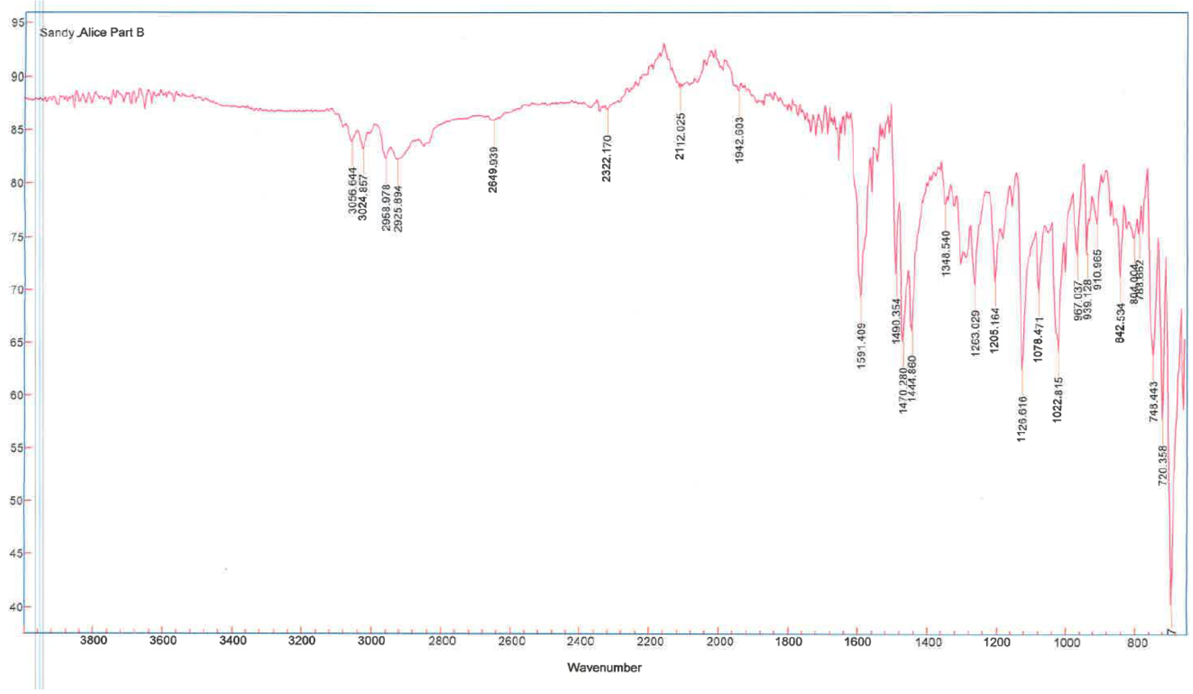

Figure S3: ATIR of compound **8**.
